# Context-specific strategies for enhancing HPV vaccination: key informant perspectives from selected U.S. jurisdictions in the post-COVID-19 era

**DOI:** 10.3389/fpubh.2026.1810928

**Published:** 2026-09-24

**Authors:** Erika Escabí-Wojna, Natalie Álamo-Rodríguez, Mariola Acosta-Rullán, Grace Vélez-Crespo, Vivian Colón-López

**Affiliations:** 1 Department of Cancer Control and Population Sciences, University of Puerto Rico Comprehensive Cancer Center, San Juan, Puerto Rico; 2 Department of Environmental Medicine and Public Health, Icahn School of Medicine at Mount Sinai, New York, NY, United States; 3 Center for Tropical Diversity, Puerto Rico Science, Technology & Research Trust, San Juan, Puerto Rico

**Keywords:** cancer prevention, HPV school entry policy, HPV vaccination, HPV vaccination strategies, qualitative research

## Abstract

**Introduction:**

Despite the availability of a safe and effective Human Papillomavirus (HPV) vaccine, uptake remains inconsistent across the United States (U.S.), with marked differences in vaccination coverage across states. This descriptive exploratory study examined key informant perspectives on HPV vaccination promotion across policy-engaged jurisdictions in the U.S. in the post-COVID-19 era.

**Methods:**

Semi-structured interviews were conducted with nine key informants across six U.S. jurisdictions. Classification of the interviews was based on those with existing HPV vaccine school-entry requirements or those jurisdictions with recent school-entry policy considerations. Using inductive thematic analysis, we synthesized key informant perspectives and organized themes using analytical dimensions informed by the socio-ecological model.

**Results:**

Findings highlight the importance of provider education, school-entry requirements, cancer prevention messaging, trusted messengers, culturally tailored outreach, and cross-sector partnerships as important components of HPV vaccine promotion. Barriers such as vaccine misinformation and increased hesitancy following the COVID-19 pandemic were also discussed.

**Discussion:**

These insights can inform targeted, context-specific strategies to improve HPV vaccination rates and reduce cancer disparities.

## 1 Introduction

HPV is the most common sexually transmitted infection in the U.S. and a leading cause of cervical and other HPV-related cancers (1). Over 49 million people in the U.S. are affected by HPV, which causes about 90% of cervical cancer cases (2). Despite the availability of an FDA-approved HPV vaccine, vaccination completion rates significantly vary by state, ranging from 39.1 to 79.8% among adolescents, leaving many vulnerable to this infection and contributing to cancer health disparities (3).

Hoss et al. (4) carried out an analysis of U.S. state statutes and regulations related to HPV vaccination. This cross-sectional study identified diverse legal strategies across 34 jurisdictions and the District of Columbia (DC), including mandates, public awareness campaigns, education initiatives, and strategic planning requirements in health department programs. As of May 2024, only DC, Virginia, Rhode Island, Hawaii, and Puerto Rico require HPV vaccination for school attendance, with varying exemption policies (5, 6). Other states promote HPV vaccination through provider recommendations and educational efforts embedded in school curricula. These jurisdictional differences suggest that vaccination strategies are shaped not only by the availability of evidence-based interventions, but also by the policy, institutional, and socio-cultural contexts in which those interventions are implemented.

The socio-ecological model is a useful framework for understanding HPV vaccination because uptake is influenced by factors operating across multiple levels, including individual and interpersonal (e.g., family beliefs), organizational (e.g., provider recommendation practices, health system capacity, and provider education), community (e.g., trust and misinformation), and policy-level (e.g., vaccine requirements and insurance) factors (7–9). Understanding the multilevel factors that shape vaccination efforts is essential for guiding effective public health strategies and policy development. Beyond documenting state-level laws and interventions, there remains a need to examine how stakeholders understand the barriers, facilitators, and real-world implementation conditions that influence HPV vaccination strategies across jurisdictions.

Despite extensive research on HPV vaccination uptake and school-entry policies, limited qualitative evidence describes how organizational, community and policy-level barriers and facilitators shape decisional process regarding HPV vaccination strategies across U.S. jurisdictions with existing or recently considered HPV school-entry requirements (8, 10). This gap is especially relevant in the current vaccination climate, where pandemic-related disruptions to routine vaccination, vaccine fatigue, and increased skepticism may have compounded existing barriers to HPV vaccine promotion (11–13). Given the limited evidence on how stakeholders perceive these interacting factors across policy-engaged jurisdictions, and how national level qualitative data can help inform future strategies, a descriptive exploratory qualitative approach was appropriate. This study addresses this gap through key informant interviews conducted across multiple U.S. jurisdictions with existing HPV vaccine requirements or those that had recently considered adopting such requirements to identify context-specific factors influencing HPV vaccination strategies and uptake, including how stakeholders perceive the continuing influence of the COVID-19 pandemic on HPV vaccination efforts.

## 2 Methods

We selected 12 U.S. jurisdictions, categorized into three groups based on their HPV vaccination policy context and comparable sociodemographic compositions. These included (1) jurisdictions with existing HPV vaccine requirements (Rhode Island, District of Columbia, Virginia, and Puerto Rico), (2) those that had recently considered adopting such requirements (Maryland, California, New York, and Massachusetts), and (3) comparable jurisdictions that had not considered requirements (Oklahoma, Mississippi, Iowa, and New Hampshire). For each jurisdiction, we identified between four to eight potential key informants (KI), resulting in a total pool of 69 individuals. Potential key informants were identified through online searches of state and jurisdictional health department websites, immunization programs and coalition webpages, and professional medical association websites. This pool represented potential key informants identified to be invited to participate in the study rather than a predetermined target sample size. These informants represented a range of roles, including immunization program managers, health department directors, representatives from pediatric and family physician associations, and state legislators. Inclusion criteria required participants to be at least 21 years old, have at least 3 years of experience in health or vaccination policy, and have knowledge on HPV vaccination strategies.

Recruitment was conducted via email with up to two follow-up reminders, directing potential participants to a REDCap screening survey. Eligible respondents were redirected to the informed consent form and asked to indicate their interview availability. Between May and August 2024, 12 individuals initiated the screening survey, 10 completed it, and nine met the eligibility criteria. Thus, the final sample reflects the eligible participants who completed recruitment and enrollment during the study period. Although the original sampling frame was developed to support comparisons across jurisdictions with different HPV vaccination policy contexts, a small final sample (*n* = 9) was recruited and did not yield participants from jurisdictions without HPV school-entry policy consideration. As a result, the final analysis was not designed to formerly compare jurisdictions with and without policy activity. Instead, the study was reframed as a descriptive exploratory thematic synthesis of key informant perspectives from jurisdictions with existing (*n* = 7) or recently considered HPV school-entry requirements (*n* = 2), offering valuable insights into common and divergent factors shaping HPV vaccination strategies. In this study, the term “multilevel” refers to the analytical organization of participants’ perspectives across provider/organizations, community, and policy/contextual dimensions informed by the socio-ecological model, rather than to sampling participants from each ecological level.

While the original intent was to compare perspectives across specific jurisdictions with varying policy environments, confidentiality concerns limited the ability to attribute findings to individual jurisdictions. To protect the identities of key informants, many of whom hold unique roles within their jurisdictions at the time of the interviews, state-level identifiers were excluded from all published materials. Because recruitment yielded only nine eligible participants, data saturation was not reached; therefore, the themes identified should be interpreted as exploratory and should not be considered as exhaustive representation of key informant perspectives across HPV vaccination policy contexts.

The semi-structured interview guide was developed using the study aims and existing HPV vaccination literature and is provided as Supplementary material 1. Questions focused on three primary domains: (1) the current HPV vaccination context within the jurisdiction, including ongoing vaccination promotion strategies; (2) perceived barriers and facilitators affecting HPV vaccination efforts; and (3) the impact of the COVID-19 pandemic on HPV vaccination promotion and implementation activities. All interviews were conducted virtually via Microsoft Teams and lasted 45 to 90 min. Research team members rotated across moderator, co-moderator, and technical support/note-taking roles. A designated note-taker documented relevant observations and contextual information during each interview. Interviews were audio-recorded with participant consent and transcribed verbatim for analysis.

An initial codebook was developed through a close review of the transcripts while remaining informed by the broad domains explored in the interview guide. The codebook was refined iteratively as new concepts emerged from the data. Verbatim transcripts were uploaded to Dedoose, where the research team applied codes to relevant excerpts and organized coded data for thematic analysis using an inductive, iterative approach. Following the consensus-based team-coding model approach outlined by Guest and colleagues (14), three trained researchers independently coded each transcript and met regularly to compare coding decisions and reach consensus. Coding discrepancies were resolved through discussion until agreement was reached.

After coding was completed, themes were organized and interpreted using analytical dimensions informed by the socio-ecological framework proposed by Ryan et al. (8), adapted from the Centers for Disease Control and Prevention (CDC) model (15). Thus, while theme generation was data-driven, the socio-ecological framework provided a structure for presenting and interpreting the findings. An overview of the study recruitment, data collection, and qualitative analysis workflow is presented in Figure 1. The protocol was approved by the Institutional Review Board of the University of Puerto Rico Comprehensive Cancer Center (#2023-04-98).

**Figure 1:**
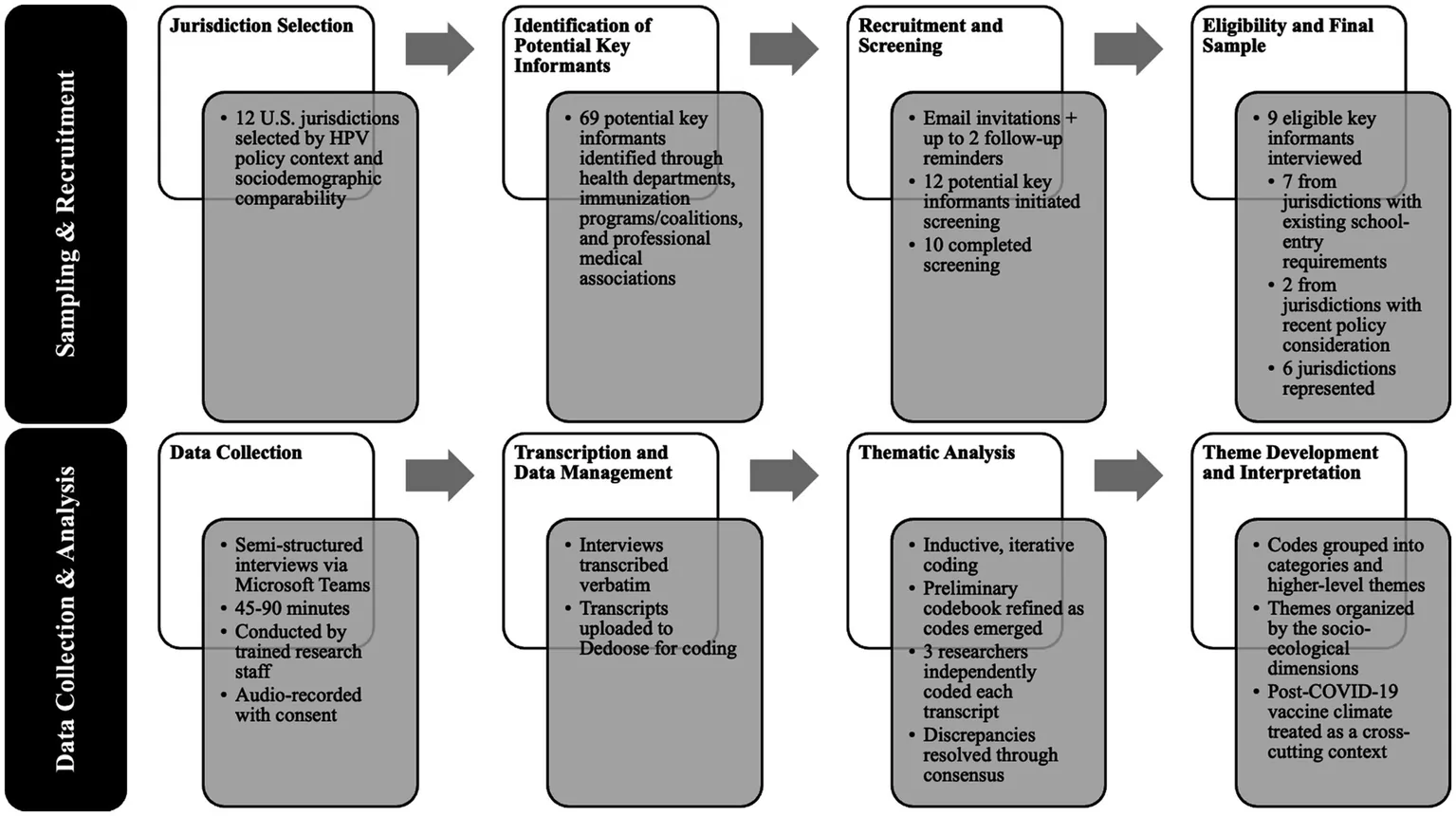
Study recruitment, data collection, and qualitative workflow.

## 3 Results

Nine key informants were recruited, representing public health professionals (*n* = 6), health educators (*n* = 2), and an epidemiologist (*n* = 1), from six jurisdictions: Puerto Rico, Virginia, District of Columbia, New York, Rhode Island, and California. Participants were 89% female, and 55.6% had five to 10 years of experience in HPV vaccination strategies. Seven out of the nine key informants were from jurisdictions with an HPV vaccine school-entry requirement.

To organize key informants’ perspectives identified in the interviews, findings were grouped into three analytical dimensions informed by the socio-ecological model: (1) provider and organizational factors, (2) community factors, and (3) policy and contextual factors. A fourth section describes the post-COVID-19 vaccine climate as a cross-cutting context influencing these dimensions. Table 1 provides representative excerpts supporting each theme identified within the provider/organizational, community, policy/contextual, and post-COVID-19 dimensions. Quotations are presented using anonymized key-informant identifiers to protect participant confidentiality.

**Table 1:** Analytic dimensions, themes, and representative key-informant excerpts related to HPV vaccination strategies, barriers, and facilitators (*n* = 9).

Analytic dimension	Theme	Excerpt
Provider and organizational factors		
	Health provider recommendation	“That cooperation from the doctor is vital, because it’s the person the parent has contact with, the one the parent trusts, right? So having them as allies is always necessary.” - KI #3
	Shift in promotional message	“[…] Using that cancer prevention angle, because the way that HPV vaccination was introduced, it was more of a, I’m not going to say promiscuity angle, but parents may have felt that “oh, if my child gets this vaccine that may give them permission to engage in sexual activity. So, that was really not the way to introduce that vaccine. So, we are looking at it and trying to change that perception by looking at it from a cancer prevention angle. And that, I think, has been really helpful.”–KI #7
	Start vaccination at age nine	“[.] A lot of effort has been put into educating about the strategy of starting the HPV vaccination series at ages 9 or 10. And I think that’s been effective because we certainly see over time that more and more providers, even though it’s still a slim minority, but more and more providers are adopting that strategy.”–KI #10
	Educational material	“But I do feel like this is an effective strategy, you know, even if it’s successful in pointing parents in the direction of more information. You know, I’ve had some really great calls with parents who called in and were on guard about, like, “why am I being told to give my seventh grader an HPV vaccine?” and through talking with them, it’s not that they have drastically changed their mind. They’re just open to listening, which, in my view, that’s a success, right? So, I find that [educational materials] can facilitate additional discussions.”–KI #8
	Dilution of HPV-specific messaging	“We do back to school campaigns, but we do not really specifically call out HPV vaccine. We just say, you know, it’s back to school, make sure your kid is up to date with all their vaccines so that they can be healthy and stay in school [.] one wonders how effective those messages can be when they are really general like that, but I do not think that they are not effective. I just do not know if they are as effective as maybe some of the more personalized efforts that we do.”–KI #16
Community factors		
	Address HPV vaccine myths/misinformation	“[A parent] had heard that the vaccine is supposedly related to infertility, when that has been disproven a lot of times, so I think we definitely have to continue making an effort to continue focusing this vaccine on cancer prevention, but also to work on all the myths that surround it.”–KI #11
	Community engagement and outreach strategies	“I think always keep in mind trusted messengers. I think that’s really important in terms of getting your message across effectively to communities and understanding that those messengers or those trusted messengers are different in different communities across the state.”–KI #10
	Misinformation/misconceptions	“You know that people do not know the boy should get it, that we are still dealing with, in questions around the vaccine causing infertility. And I’m like, did not we deal with all of this? Like, when the vaccine first came out. So, I think it’s just reiterating that message.”–KI #16
	Groups against HPV vaccination	“I cannot think of, off the top of my head, any organizations, but I know they do exist. So, I would say general, you know, anti-vaxx groups would be against this particular vaccine. [.] I do believe that spreading of misinformation hinders our efforts because anti-vaxxers are very vocal. They’re very loud. They have the ability to really get information out, get the attention of people because they are loud. They’re a mighty group.”–KI #7
	Groups in favor of HPV vaccination	“[Collaborators] are good, not just for this vaccine, for all vaccines. Medical schools too. Since COVID, medical and public health schools have also become a bit more active and more vocal in helping, promoting, and maintaining the general concept of vaccination, again, not just the HPV vaccine–all vaccines.”–KI #12
	Institutional support	“Our huge convener of community partners is our immunization coalition. And when you see the number of partners that participate in that coalition, it’s really reassuring because it reminds you that there’s just a diversity, there’s a diverse number of professionals in different fields who are advocating for immunizations in general, including HPV. So, yes, I would say that we are very well supported by our community-based organizations.”–KI #8
Policy and contextual factors		
	Political facilitators	“Another thing with the HPV vaccine being a school-required vaccine, it makes it immensely accessible, you know, especially given how expensive it is. So, you know, under and uninsured adolescents are able to go to a VFC provider and they are able to get that shot.”–KI #8
	Political barriers	“In the legislature, there are groups now that were not present before, and they are definitely anti-vaccine. They are conservative, and within their philosophy, they try to present their objection to the use of that type of vaccine based on their beliefs.”–KI #3
	Cultural facilitators	“We do not really have a huge anti-vax movement up here. So, people for the most, I mean, not to say we do not have them. We definitely have a few, very vocal, frequent fliers. But it’s not nearly at the level of some of my other colleagues have to deal with.”–KI #16
	Cultural barriers	“Some of the common themes, which I think I’ve heard from partners who are in similar spaces as I am, it’s the transmission- the fact that parents feel that it’s an inappropriate vaccine for an 11-year-old, and because of the fact that you cannot catch it in school.”–KI #8
	Access to health services	“[.] If you have coverage, if you do not have coverage, that greatly affects whether people have access to the vaccine.”–KI #3
	Mandate opposition	“I think we have a strong movement in a lot of jurisdictions in our state, but you know, almost exclusively rural areas of the state, not wanting to see government interference in their day-to-day lives, including health decisions, health recommendations.”–KI #10
	HPV vaccine policy perception	“I would say the majority are in interest. I think, you have those that, you know, strongly support vaccinations, then you have those that support vaccinations, but do not agree with there being maybe policies, laws, requirements for vaccines. And then you have those that are like staunchly opposed. And obviously there’s, you know, there’s a continuum. So, there are people in between those main categories. But I would say overall our general community supports vaccination, supports HPV vaccination.” - KI #10
COVID-19 impact on vaccination efforts		
	Accessibility	“And I think all those things, lockdown, the physical distancing, trying to get an appointment, you know, a safe appointment, you know, they probably were only going to their doctors when they were feeling sick. So, yeah, I think that all of that impacted HPV coverage rates.”–KI #9
	Not a priority	“In general, much of what was done for COVID-19 prevention ended up perhaps affecting some other (vaccine) coverage because people were more focused on COVID vaccination and COVID prevention, right, all the strategies of staying at home, not socializing, right, randomly with just anyone at any time, were used.”–KI #11
	Impact of the COVID-19 vaccine	“I think that there’s probably more resistance, but that’s probably because we are seeing a greater resistance to all vaccines since the COVID pandemic. And with all of the hesitancy and lack of confidence in vaccines during the pandemic, I think it has affected vaccines in general.”–KI #9

### 3.1 Provider and organizational factors shaping HPV vaccination

Various effective strategies to improve HPV vaccination rates were reported by key informants. Notably, healthcare provider recommendation and training providers to recommend the vaccine to parents of eligible children were stressed as crucial in boosting HPV vaccination, with participants stating that parents typically trust them. They also emphasized the importance of reframing HPV vaccination from sexually transmitted infection (STI) prevention to cancer prevention. One key informant noted that early HPV vaccine rollout was negatively impacted by concerns of sexual promiscuity, but shifting the messaging to cancer prevention has been very helpful. Overall, participants perceived that normalizing the vaccine as a routine health behavior could improve parental acceptance. Providers should be equipped not only with accurate information about the vaccine, but also with the communication skills needed to explain why HPV vaccination is important, recommend it confidently to parents, and respond to common myths and misinformation. In this sense, provider education is a good starting point in improving the quality and consistency of provider recommendation.

Other effective strategies that were reported included starting HPV vaccination at age 9 and providing culturally tailored educational materials, such as factsheets, to help initiate conversations between parents and providers. Key informants were hesitant to signal any one vaccination strategy as ineffective. They mainly expressed that HPV vaccination promotion tends to get diluted among other vaccines in their vaccination campaigns, as there are not as many efforts for the HPV vaccine alone. This suggests that general vaccination campaigns may be perceived as less useful for HPV vaccine promotion when they do not directly address HPV-specific concerns, such as cancer prevention or misinformation.

### 3.2 Community factors shaping HPV vaccination

Key informants also reported the importance of using trusted community messengers and culturally tailored outreach to promote the HPV vaccine. One participant emphasized the need for linguistically and culturally appropriate outreach, taking into consideration how different communities within their same jurisdiction require different trusted messengers and approaches. Some verbalized that it is important to work with community-based organizations, religious leaders, and local advocates to build trust and combat hesitancy.

Misinformation about the HPV vaccine emerged as a common barrier for promotion efforts. Key informants shared similar concerns about prevalent myths, such as the belief that the vaccine causes infertility or that it is intended only for females. They pointed to social media as an amplifier of these myths by anti-vaccine groups that “recycle the same arguments,” which leads parents to repeatedly “catch wind of the same myths.” Participants’ accounts suggest that misinformation operated within a broader informational and socio-political environment shaped by social media, anti-vaccine messaging, cultural concerns about adolescent sexuality, religious objections, and resistance to government involvement in health decisions. In this context, trusted messengers and culturally tailored outreach can help respond to misinformation and myths in ways that are community specific. These findings also connect to the provider and organizational dimension, as participants described provider education as an opportunity to address misinformation during parent-provider interactions and reinforce HPV vaccination as cancer prevention.

Key informants consistently highlighted institutional support as an important facilitator of HPV vaccine promotion. This support included immunization coalitions, community-based organizations, academic institutions, medical associations, and parent-led advocacy organizations that helped sustain and coordinate vaccination efforts. Participants emphasized that the diversity of collaborators strengthened promotion, underscoring the importance of cross-sector and multisectoral collaboration.

### 3.3 Policy and contextual factors shaping HPV vaccination

Participants from jurisdictions with school-entry requirements perceived these policies as having contributed to higher HPV vaccine uptake. One key informant even stated that adding HPV to the list of required vaccines helped persuade hesitant parents (or those “on the fence”) to get their children vaccinated. This requirement was described as the primary political facilitator for vaccine promotion.

Key informants also identified policy and structural barriers. These included political barriers, such as opposition from government figures or political groups to vaccination mandates and efforts to make existing requirements more flexible. Structural barriers were also noted, particularly limited access due to gaps in insurance coverage or providers lacking vaccine supply. Cultural barriers emerged as well, including concerns that vaccination encourages sexual promiscuity and resistance from some religious communities. Participants also referenced opposition to government involvement in health decisions, which they perceived as constraining HPV vaccine promotion and the adoption of school-entry requirements in jurisdictions without the policy.

The majority of key informants expressed that overall, there is an understanding of the importance of vaccination policies, but there are varying degrees of opposition to the HPV school-entry requirement. One participant clarified that resistance was directed more toward the mandate itself than toward other HPV-related initiatives, such as parent education. Several participants observed that parents are attentive to meeting back-to-school requirements and, when applicable, exploring opt-out clauses. Informants from jurisdictions without a school-entry requirement described limited political will to pursue such legislation. One key informant explained that their state currently has “little appetite for legislation,” making the enactment of an HPV school-entry policy unlikely and noting considerable opposition to establishing the mandate. Another participant emphasized that this resistance does not necessarily stem from vaccine hesitancy, but rather from broader objections to vaccine mandates.

Together, these findings suggest that policy context shaped HPV vaccine promotion in two ways. Participants’ accounts suggest that, in jurisdictions with school-entry requirements, these policies may support normalization and access. In jurisdictions with recent policy consideration but no requirement, participants described political resistance and limited legislative appetite as constraints on mandate adoption, making non-mandate strategies such as education, early vaccination, and provider engagement especially important.

### 3.4 COVID-19 impact on vaccination efforts

Key informants consistently noted that the COVID-19 pandemic disrupted access to routine vaccinations, as healthcare systems prioritized COVID-19 immunization once it became available. Some participants observed a shift in emphasis toward respiratory vaccines, such as influenza and RSV, which they attributed to heightened awareness following the pandemic. Key informants also highlighted that the introduction of the COVID-19 vaccine prompted broader questioning of vaccine efficacy and contributed to increased hesitancy and distrust. One participant suggested that this hesitancy was not resistance to vaccination but reflected fatigue with ongoing vaccine-related discourse: “The [COVID-19] vaccine has affected parents’ perceptions of all vaccines, and that would include the HPV vaccine […] there was this added layer of distrust in vaccines following COVID-19 vaccine mandates.” Interestingly, perspectives varied, with some key informants describing a polarizing effect, strengthening confidence in vaccines for certain individuals while amplifying doubts among others.

Participants’ accounts suggest that the COVID-19 pandemic intensified existing challenges related to routine vaccination and vaccine confidence. Pandemic-related disruptions affected access to routine preventive care, while vaccine fatigue and distrust complicated communication about HPV vaccination. Provider recommendation, community trust, and clear cancer prevention messaging become especially important for maintaining confidence in HPV vaccination.

## 4 Discussion

This descriptive exploratory study identified multilevel factors shaping HPV vaccination promotion strategies across U.S. jurisdictions with existing or recently considered HPV school-entry requirements. At the provider and organizational level, findings were consistent with prior research where provider recommendation (16–21), provider education to recommend the vaccine (16–20) and reframing HPV vaccination as cancer prevention (16, 18–20) emerged as important strategies for vaccine uptake and promotion. Key informants repeatedly described clinicians as trusted sources of vaccine information for parents, and, therefore, strengthening the provider role remains one of the most effective strategies for improving coverage. Provider education could be a foundational strategy because it prepares clinicians not only to understand the vaccine, but also to recommend it confidently, communicate its importance to parents, and address HPV vaccine myths or misinformation during clinical encounters. Taken together with prior research, these key informant perspectives suggest that provider recommendation may function as a bridge between health system strategies and parent-level acceptance, particularly when HPV vaccination is framed as routine cancer prevention rather than STI prevention.

At the community level, key informants identified misinformation as an important barrier to HPV vaccine promotion. They also emphasized trusted messengers and culturally tailored communication as strategies for addressing vaccine concerns within communities. Although informational factors are not a distinct level in the socio-ecological model, they operated as a cross-cutting feature of the vaccination environment in this study. Participants’ descriptions of misinformation, including its amplification through social media, are consistent with previous studies (18–23). Their accounts suggest that misinformation should be understood as part of a broader environment shaped by anti-vaccine messaging, circulating myths, and the communication channels through which parents encounter vaccine-related information. These findings underscore the importance of culturally tailored educational strategies, trusted community messengers, and enhanced provider training to address parental concerns described by key informants in ways that are locally credible and responsive to the specific narratives circulating within communities.

Key informants also emphasized cross-sector collaboration, including partnerships with immunization coalitions, community-based organizations, academic institutions, medical associations, and parent advocacy groups, consistent with previous research (16, 18, 20). These collaborations may expand the reach of vaccination efforts while supporting culturally appropriate outreach and responses to misinformation.

At the policy and contextual level, school-entry vaccination requirements were also identified as powerful structural drivers of uptake, with participants reporting that these requirements normalized HPV vaccination, improved access, and even persuaded hesitant parents. These findings are consistent with prior research demonstrating that school-entry HPV vaccination policies, particularly when combined with other strategies (24), improve uptake (17, 25).

Moreover, qualitative studies have shown that the absence of school entry requirements has emerged as a key barrier to HPV vaccination (18, 20, 26). Consistent with prior literature, key informants described structural barriers, such as inconsistent insurance coverage, limited provider supply (16, 18, 20, 24, 27) and cultural concerns about sexual behaviors (18, 20, 21, 26), as factors they perceived to influence HPV vaccination decisions. Expanding the number of providers enrolled in the Vaccines for Children (VFC) program may help address barriers in access to the HPV vaccine by ensuring consistent vaccine availability and reduce financial obstacles (16, 20, 27).

Although formal comparisons across policy contexts were not possible, participants from jurisdictions without school-entry requirements emphasized non-mandate strategies such as early vaccination and educational outreach. Their accounts also suggested that resistance was often directed toward mandates rather than the HPV vaccine itself, reflecting broader concerns about government involvement in health decisions. Consistent with McKeithen et al. (27), these findings suggest that the effectiveness of school-entry requirements may depend on careful policy design that reflects local context, as these policies may either be too weak to change behavior or too controversial to be successfully implemented.

Participants described how the COVID-19 pandemic disrupted routine preventive care, redirected health system attention towards COVID-19 and other respiratory vaccines, and intensified vaccine fatigue, skepticism, and distrust, affecting both service delivery and the broader information environment in which HPV vaccination messages were received. As a result, post-COVID-19 HPV vaccination strategies may need to address not only missed routine vaccination opportunities, but also broader shifts in vaccine confidence and public trust.

This study has several limitations. Although a qualitative study, the small sample size and absence of key informants from jurisdictions without HPV school-entry policy consideration limit the breadth of the findings and preclude formal comparisons across policy contexts. The final sample may also reflect greater interest or engagement with HPV vaccination among those who participated. Nevertheless, the inclusion of key informants from six jurisdictions provided information-rick perspectives on vaccination strategies, barriers, facilitators, and post-COVID-19 challenges. Findings should therefore be interpreted as preliminary and exploratory rather than representative of all U.S. jurisdictions.

Several steps were taken to strengthen the dependability and transparency of the findings. Research team members completed shared training in interview guide development, qualitative interviewing, codebook development, and thematic analysis, and conducted practice interviews before data collection. During analysis, the team used a subjective agreement approach to assess intercoder agreement, consistent with Guest et al. (14). The team also maintained an audit trail documenting who was involved in each stage of the research process, including moderation, co-moderation, note-taking, transcription, coding, and analysis. These procedures helped reduce individual coder bias and strengthen the credibility of the thematic interpretation.

Future research should build on these exploratory findings through comparative studies that intentionally recruit jurisdictions with and without HPV school-entry requirements, as well as jurisdictions with no recent policy consideration. Mixed method designs that integrate key informant perspectives with HPV vaccination coverage data could help assess how perceived barriers and facilitators align with measurable vaccination outcomes. Future studies could also examine how post-COVID-19 vaccine confidence, routine vaccination patterns, and policy attitudes evolve over time.

In conclusion, this study provides exploratory qualitative insights into HPV vaccination promotion across six U.S. jurisdictions with existing or recently considered HPV school-entry requirements. The findings highlight the importance of tailoring HPV vaccination strategies to local policy, organizational, and community contexts, particularly in an environment shaped by misinformation, mandate opposition, access barriers, and changing vaccine confidence. While findings are not generalizable to all jurisdictions, especially those without prior HPV school-entry policy consideration, they offer key informant insights that may help guide future research and context-specific strategies to strengthen HPV vaccination promotion and reduce HPV-related cancer disparities.

## Data Availability

The raw data supporting the conclusions of this article will be made available by the authors, without undue reservation.
