# Supplementary material for: Context-specific strategies for enhancing HPV vaccination: key informant perspectives from selected U.S. jurisdictions in the post-COVID-19 era
Source: Front Public Health. 2026 Sep 24;14:1810928. doi: 10.3389/fpubh.2026.1810928 (PMC13646127; doi:10.3389/fpubh.2026.1810928)
Supplement: Supplementary file 1 [file Table_1.DOCX]

**Supplementary Material 1. Key Informant Interview Guide**

I. **Sociodemographic information**
   1. What type of organization do you work for?
      i. Government
      ii. Public entity
      iii. Education
      iv. Private entity
      v. Other, specify:
   2. What is your profession?
      i. Physician, specify specialty:
      ii. Public Health
      iii. Researcher/Investigator
      iv. Nurse
      v. Administrator
      vi. Health Educator
      vii. Data Analyst
      viii. Pharmacist
      ix. Legislator
      x. Other, specify:
   3. What is your job title?
   4. How many years of experience do you have in your current role?
      i. Less than 5 years
      ii. 5-10 years
      iii. 11-15 years
      iv. More than 15 years
   5. What state/territory do you reside in?
      i. California
      ii. District of Columbia
      iii. Iowa
      iv. Maryland
      v. Massachusetts
      vi. Mississippi
      vii. New Hampshire
      viii. New York
      ix. Oklahoma
      x. Puerto Rico
      xi. Rhode Island
      xii. Virginia
II. **Current state of HPV vaccination**
    1. What is the current HPV vaccination rate in your state?
       xiii. Variation in vaccination rates by ethnic groups, sociodemographic status, etc.
    2. What strategies, if any, have been implemented by your organization to increase HPV vaccination rates? (e.g., educational campaigns, community outreach, public policy) How long have these strategies been in place?
    3. If your organization has developed a message for promoting or educating about the HPV vaccine, can you tell us a little about it? E.g., for what purpose it was created, what audience it is aimed at, etc.)
    4. What strategies have been effective in increasing HPV vaccination rates?
       i. What have been the major successes of HPV vaccination strategies in your jurisdiction?
          1. If possible, please provide examples, data, or evidence of these successes.
    5. Which ones have not been effective?
       i. Could you give us examples?
    6. As a result of the strategies implemented by your organization, have you observed any changes in the levels of vaccine acceptance in your state? Please elaborate.
    7. Is your organization developing any additional strategies that will be implemented in the future?
       i. Is there a strategic plan in place?
       ii. When do you plan to implement it?
    8. From your perspective, what strategies do you consider could be effective to promote HPV vaccination given the socioeconomic, political and cultural context of your jurisdiction?
III. **Challenges and facilitators**
     1. In addition to the COVID-19 pandemic, what have been the political, socioeconomic, and/or cultural factors that have affected HPV vaccination efforts in your state?
     2. In addition to the COVID-19 pandemic, what have been the political, socioeconomic, and/or cultural factors that have facilitated vaccination efforts in your state?
     3. What organizations do you know that are in favor of the HPV vaccine?
        i. Do you receive support from them?
           1. If you receive support, what type?
     4. What types of organizations or groups are against the HPV vaccine?
        i. To what extent do they hinder your organization's efforts to increase HPV vaccination? What strategies do they use?
     5. Is there interest or opposition from the general community regarding HPV vaccination policies (e.g., vaccination requirement for school entry, distribution of educational materials, including information about the HPV vaccine in the school curriculum) in your jurisdiction?
        i. Regarding these policies, could you tell us if there is interest or opposition from the medical community?
           1. Ex. Pediatricians, OB-GYNs, dentists, etc.
IV. **COVID-19 impact**
    1. What impact, if any, did the strategies put in place to mitigate the spread of COVID-19 have on HPV vaccination efforts? (e.g. Lockdown, physical distancing, coordination of medical appointments, mobilization)
       i. Were there any HPV-related strategies that had to be stopped due to the challenges caused by the pandemic? Has there been an increase or decrease in HPV vaccination rates? Please elaborate.
       ii. Do you think there is greater or lesser resistance to HPV vaccination? Please elaborate.
    2. How do you compare public health communication of the COVID-19 vaccine and the HPV vaccine?
    3. How do you compare access to the COVID-19 vaccine and the HPV vaccine?
    4. How do you compare the community response to the COVID-19 vaccine and the HPV vaccine?
    5. How did the introduction of the new COVID-19 vaccine affect parents' perceptions of the HPV vaccine?
       i. Has it been beneficial?
       ii. Has it been a hindrance?
    6. In your experience, what decision are parents making regarding vaccine bundling?
       i. Ex. Do parents prefer to administer the 3 recommended/required vaccines for their children at age 11 (HPV, meningococcus (MenACWY), and tetanus, diphtheria, and pertussis (Tdap)) during the same appointment, or do they prefer to separate them?
          1. Does this decision vary by race/ethnicity?
       ii. What do you think about recommending the HPV vaccine from age 9 instead of age 11?
    7. At an institutional level, in what order of priority is HPV vaccination after the COVID-19 emergency? Please elaborate.
